# Explorative case control study on the associations of serum vitamin D3, folic acid and vitamin B12 levels on Kawasaki disease and coronary artery lesions

**DOI:** 10.3389/fnut.2024.1402316

**Published:** 2024-06-11

**Authors:** Yanfei Chen, Xingzhu Liu, Bin Li, Jun Li, Lijuan Meng, Caixia Ye, Linfei Han, Hong Li, Li Li Deng, Zhongjian Su, Xing Zhang

**Affiliations:** ^1^Kunming Children’s Hospital, Kunming, China; ^2^Department of Immunology, Center of Immunomolecular Engineering, Innovation and Practice Base for Graduate Students Education, Zunyi Medical University, Zunyi, China; ^3^Maternity and Child Health Care Hospital of Yunyang County, Chongqing, China

**Keywords:** Kawasaki disease, coronary artery lesions, folic acid, vitamin D3, vitamin B12

## Abstract

**Background:**

Kawasaki Disease (KD) is a pediatric vasculitic disorder characterized by systemic small vasculitis, notably coronary arteritis, with unclear pathogenesis. This explorative case-control study investigated the association between folic acid (FA), vitamin D3 (VD3), and vitamin B12 (VB12) levels and the different types of Kawasaki Disease, as well as the incidence of coronary artery lesions (CALs).

**Methods:**

In this explorative case control study, 365 KD children admitted to our hospital from January 1, 2022 to June 30, 2023 were included as the KD group. Simultaneously, 365 healthy children who received physical examination during the same period were included as the control group. The KD group was divided into typical KD group and incomplete KD group (IKD group), CALs group and non-CALS group, and IVIG sensitive group and IVIG resistant group. The children with CALs were divided into small tumor group, medium tumor group and large tumor group. Serum levels of FA, VB12, and VD3 were compared across all groups.

**Results:**

Serum levels of FA and VD3 were significantly decreased in both the KD and CALs groups (*p* < 0.05), and both factors were identified as independent risk factors for KD and CALs. Similarly, reduced serum VD3 levels were observed in the IKD and IVIG-resistant groups (*p* < 0.05), with VD3 also being an independent risk factor for both IKD and IVIG resistance. Additionally, lower serum FA levels were noted in the group with large aneurysms (*p* < 0.05), establishing FA as an independent risk factor for aneurysm size.

**Conclusion:**

Serum levels of folic FA and vitamin VD3 were significantly reduced in children with KD. Furthermore, these reductions were more pronounced in children with IKD and CALs. This pattern suggests that lower FA and VD3 levels may increase the risk of more severe coronary lesions in KD patients. Therefore, monitoring these biomarkers could provide valuable insights for early clinical diagnosis and intervention.

## 1 Introduction

Kawasaki disease (KD) is a systemic vasculitic disorder commonly seen in pediatric clinics, primarily affecting children aged 6 months to 5 years (1). The primary pathological manifestation of KD is systemic vasculitis, with coronary arteritis being the predominant form. Notably, approximately 20% of KD patients develop complications involving coronary artery lesions (CALs) (2, 3). CALs secondary to KD are linked to alterations in specific biomarkers. These include counts of platelets and neutrophils, measures of plateletcrit and platelet distribution width, along with mean platelet volume, erythrocyte sedimentation rate, cardiac troponin I, endothelin-1, albumin, and hemoglobin levels (4). In a retrospective study involving 113 Chinese children, the Kobayashi score was applied to predict CALs in KD. Although the score demonstrated predictive capabilities, its accuracy was not sufficiently high. Therefore, there is a great need for reliable laboratory markers to accurately predict the subsequent development of CAL in the acute early stages of KD (5). The pathogenesis of KD is still not completely clear. It is currently believed that KD results from abnormal immune activation, which is induced by infections, especially in individuals with genetic susceptibility (4, 6). Previous study has shown that hyperhomocysteinemia can induce subclinical endothelial dysfunction, leading to advanced coronary arteritis in patients with KD (7). 5-methyltetrahydrofolate (5-MTHF) is the main circulating form of FA and is a methyl donor for the remethylation of homocysteine (Hcy) to methionine. FA other study has indicated that 10-methylenetetrahydrofolate reductase (MTHFR) is a candidate gene involved in the process of arteriosclerosis, and MTHFR is an enzyme that catalyzes the reduction of 5, 10-methyltetrahydrofolate to 5-MTHF (8). Therefore, it can be hypothesized that folic acid (FA) levels in the body may influence the development of arteriosclerosis. However, the specific role of FA in the progression of arteriosclerosis in KD patients remains unclear. Vitamin D3 (VD3) is an essential fat-soluble vitamin and a crucial steroid hormone. Its deficiency is widespread globally. Chen et al. (9) discovered that, besides regulating calcium and phosphorus metabolism, VD3 also influences immune disorders through the modulation of inflammatory signaling pathways. Reports suggested that adjunct therapy with 1-alpha, 25-dihydroxyvitamin D3, known for its anti-inflammatory and immunomodulatory properties, is beneficial in managing KD-induced vasculitis (10, 11). Comparatively, KD patients exhibit higher Vitamin D Receptor (VDR) expression in T cells than children with febrile respiratory infections or healthy individuals, suggesting that overactivated T cells may trigger the release of 25-(OH) D3. As a result, the enhanced inflammatory response observed in patients with CALs could result in an increased expression of VDR and consequently, elevated levels of 25-(OH) D3. Moreover, vitamin D deficiency has been recognized as an independent risk factor for arterial diseases (12). Therefore, the correlation between KD and VD3 has garneredincreasing attention. One study demonstrated that circulating 25-hydroxyvitamin D3 [25-(OH) D3] could predict CALs secondary to KD in children; however, the study was limited by a small sample size and low statistical power (13). VB12 is one of the most important B vitamins and serves as the primary coenzyme of Hcy forming methionine. The reduction of elevated homocysteine levels in the body depends on both FA and VB12, which provide methyl groups enabling the remethylation of homocysteine back to methionine, thus lowering Hcy levels (14). Many researchers’ studies have demonstrated relations of high Hcy levels with low intake of FA and VB12 (15–18). FA and VB12 play important roles in remethylation and transsulfuration pathway of Hcy metabolism. A defect in any of these can lead to increased levels of homocysteine in circulation. Therefore, we hypothesized that FA, VD3, and VB12 levels are involved in the pathogenesis of KD and CALs. In this study, we used our data to conduct a preliminary exploration of the associations of FA, VB12, and VD3 on different types of KD and CALs.

## 2 Materials and methods

### 2.1 Object of study

In this prospective case-control study, 365 children with KD admitted to our hospital from January 1, 2022, to June 30, 2023 were selected as the KD group. Simultaneously, a total of 365 children from the same period were selected as the control group. The KD group was divided into typical KD (*n* = 293) and incomplete KD (IKD) (*n* = 72) groups. Based on their sensitivity to IVIG, they were divided into IVIG-sensitive (*n* = 303) and IVIG resistance groups (*n* = 62). Additionally, they were divided into CALs (*n* = 18) and non-CALs (*n* = 347) groups according to the presence of CALs, and the CALs group was further divided into small, medium, and large tumor groups according to the aneurysm size (see Table 1).

**Table 1:** Distribution of samples.

Group	*n*	Gender (male%)	Age (x̄±s)
KD	365	198 (54.25)	8.39 ± 0.62
Health group	365	188 (51.51)	8.85 ± 0.67
KD	293	160 (54.61)	8.28 ± 0.56
IKD	72	38 (52.78)	8.48 ± 0.61
IVIG-sensitive	303	164 (54.13)	8.17 ± 0.45
IVIG-resistance	62	36 (58.06)	8.22 ± 0.48
CALs group	18	11 (61.11)	8.83 ± 0.66
Non-CALs	347	148 (42.65)	8.49 ± 0.53
Small tumor	15	9 (60.00)	8.28 ± 0.55
Medium-large tumor	3	3 (100.00)	8.92 ± 0.74

### 2.2 Inclusion and exclusion criteria

The inclusion criteria were as follows: (1) a diagnosis of KD according to the 2017 American Heart Association (AHA) criteria; (2) all children in the KD group were diagnosed for the first time. (3) diagnostic criteria for coronary artery lesions (12): The Z-score was adopted as the diagnostic criterion for coronary artery lesions. (i) Normal: *Z* values < 2. (ii) Coronary artery dilation: 2 ≤ *Z* value <2.5. (iii) Small coronary aneurysm: 2.5 ≤ *Z* value < 5. (iv) Medium coronary aneurysm: 5 ≤ *Z* value < 10, absolute diameter < 8 mm. (v) Large coronary aneurysm: *Z*-value < 10 or internal diameter > 8 mm. *Z*-value ≥ 2.0 indicates CAL+. (4) IVIG resistant KD is defined as patients with KD develop recrudescent or persistent fever (*T* ≥ 38°C) at least 36 h after the end of IVIG infusion (2 g/kg).

The exclusion criteria for this study were as follows: (1) combined bacterial and viral infections; (2) combined cardiovascular and respiratory diseases; (3) use of glucocorticoids, immunosuppressants, and gamma globulin in the past 2 weeks. (4) Incomplete clinical data and unwillingness to participate with treatment.

### 2.3 Research method and observation index

Fasting venous blood (2 mL) was collected from all children and allowed to stand for 30 min before undergoing low-speed centrifugation at 2,500 rpm. After 15 min of centrifugation, supernatants were collected. Serum levels of FA (ng/ml), VD3 (VD3 refers to 1,25-dihydroxyvitamin D3, ng/ml), and VB12 (μg/l) FA were measured using an enzyme-linked immunosorbent assay (ELISA) purchased from Sigma. The levels of serum FA, VB12, and VD3 were also measured using a test kit from Shenzhen Teled Medical Co., Ltd., with an automatic chemiluminescence analyzer, VIT700.

### 2.4 Statistical approach

Statistical analyses were performed using SPSS software version 26.0. Measurement data that conformed to a normal distribution were presented as mean ± standard deviation, and analyzed using independent *T*-tests. Data not following a normal distribution were described using the median and interquartile range, and analyzed with the Mann–Whitney U test. Categorical data were expressed as percentages and assessed using the chi-square (χ^2^) test or Fisher’s exact test, as appropriate. Statistical significance was established at *p* < 0.05.

## 3 Results

### 3.1 Serum levels of FA, VB12, and VD3 among different groups

The KD group was divided into typical KD group and IKD group, CALs group and non-CALS group, IVIG sensitive group and IVIG resistant group, small tumor group, medium and large tumor group. Serum levels of FA, VB12, and VD3 were compared among all groups (see Table 2).

**Table 2:** Serum levels of FA, VB12, and VD3 among different groups (x̄±s).

Group	*n*	FA (ng/ml)	VD (ng/ml)	WB12 (ug/l)
KD	365	9.82 ± 3.81	23.55 ± 7.85	465.93 ± 170.57
Health group	365	16.01 ± 7.07	56.38 ± 14.74	501.95 ± 177.41
KD	293	12.32 ± 4.58	40.44 ± 5.55	526.65 ± 126.12
IKD	72	11.58 ± 4.33	11.81 ± 3.86	518.83 ± 121.30
IVIG-sensitive	303	16.30 ± 6.89	26.12 ± 5.73	219.25 ± 80.34
IVIG-resistance	62	13.73 ± 8.12	11.02 ± 3.58	205.31 ± 79.71
CALs group	18	4.39 ± 5.80	6.61 ± 3.02	95.51 ± 26.88
Non-CALs	347	16.61 ± 6.60	25.03 ± 8.26	106.38 ± 27.23
Small tumor	15	5.15 ± 0.85	12.88 ± 4.54	105.61 ± 4.54
Medium-large tumor	3	0.60 ± 0.00	14.03 ± 4.71	100.00 ± 3.88

### 3.2 Comparisons of FA, VD3, and VB12 levels in healthy control group and KD group

**Figure 1:**
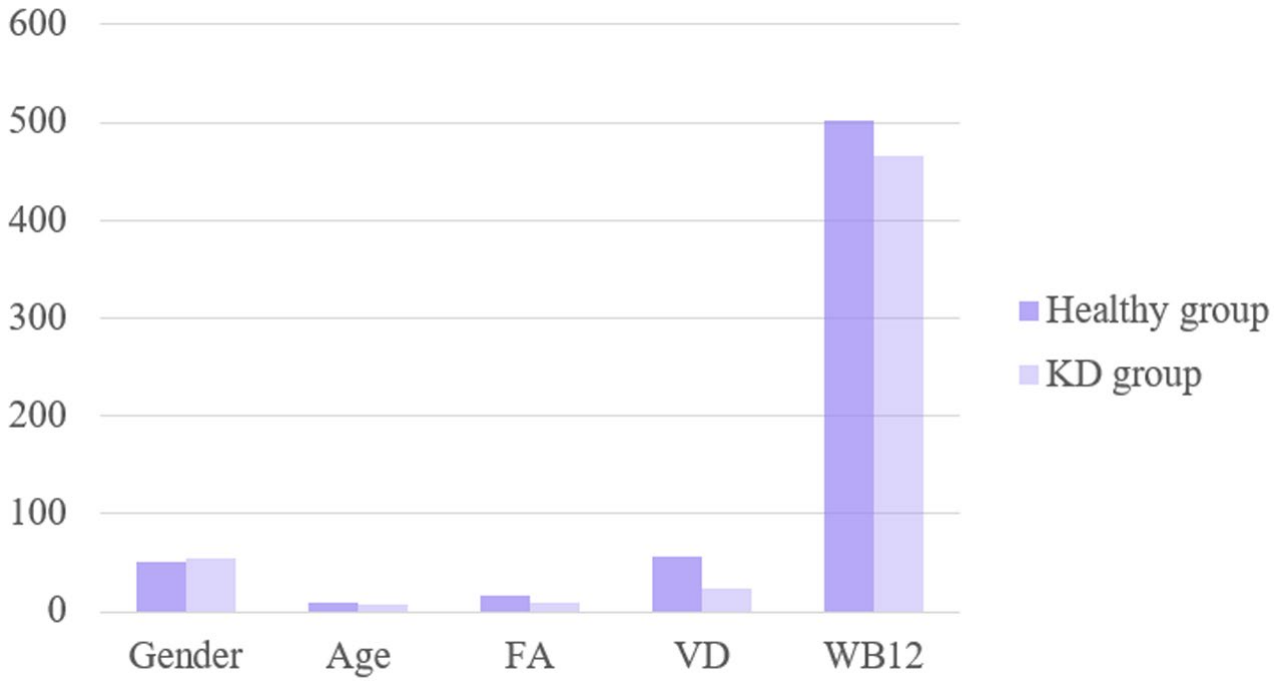
The levels of FA, VD and WB12 in healthy group and KD group were compared.

There were no significant differences in sex or age between the two groups (*p* > 0.05). The levels of serum FA and VD3 indices in the KD group were significantly lower compared those in the healthy control group (*p* < 0.05), while there was no difference in the VB12 indices between the two groups, as shown in Figure 1. Multifactor regression analysis revealed that lower FA and VD3 indices are independent risk factors for KD, as detailed in Table 3.

**Table 3:** Multiple regression analysis.

Parameters	β	SE	Wald χ^2^	OR	95%CI	*p*
FA	−1.155	0.413	7.874	3.176	1.415–7.123	0.004
VD3	−1.125	0.496	5.183	3.088	1.171–8.141	0.024
VB12	−1.334	0.290	7.992	3.345	1.515–6.995	0.089

FA, folic acid; VD3, vitamin D3; VB12, vitamin B12.

### 3.3 Comparisons of FA, VD3, and VB12 levels in typical and atypical KD groups

**Figure 2:**
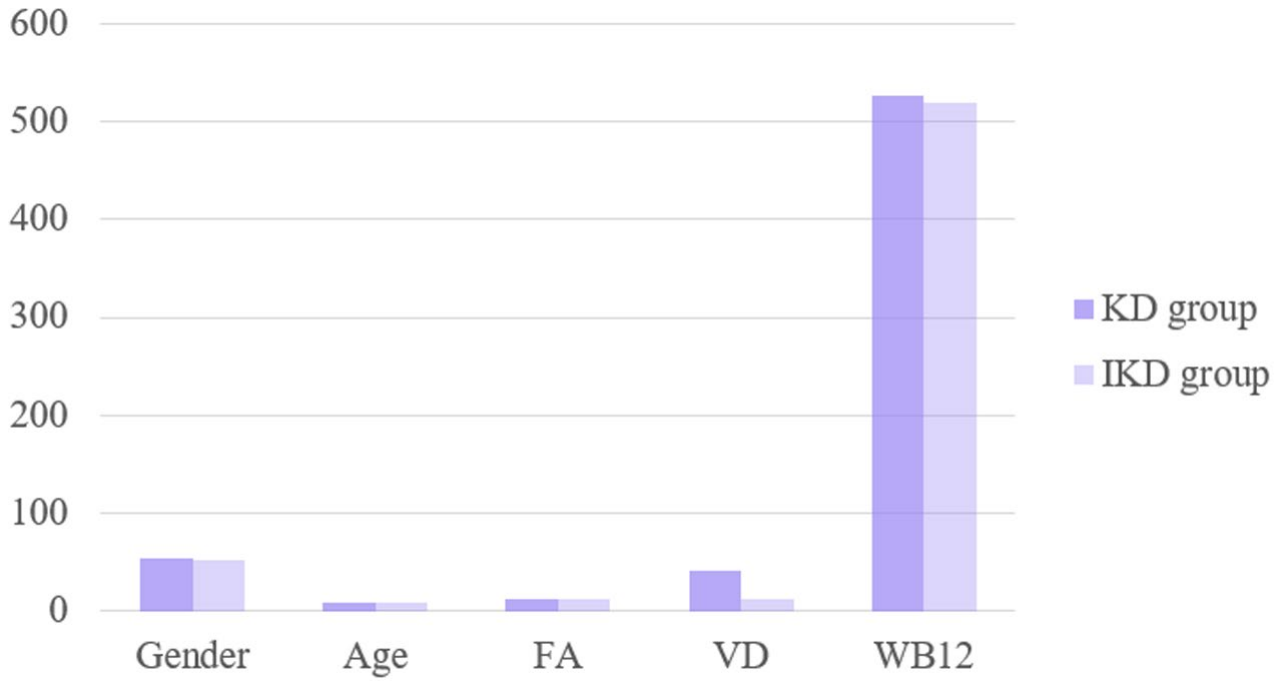
The levels of FA, VD and WB12 in KD and IKD groups were compared.

There were no significant differences in sex or age between the two groups (*p* > 0.05). Serum VD3 levels were significantly lower in the KD group compared to controls (*p* < 0.05); while there was no difference in the VB12 indices between the two groups, as shown in Figure 2. Multifactor regression analysis showed that the VD3 levels is an independent risk factor for IKD (Table 4).

**Table 4:** Multiple regression analysis.

Parameters	β	SE	Wald χ^2^	OR	95%CI	*p*
VD3	1.184	0.344	7.628	3.918	1.525–7.324	0.002
VB12	1.557	0.401	10.032	4.643	1.326–8.243	0.075

VD3, vitamin D3; VB12, vitamin B12.

### 3.4 The levels of FA, VD3, and VB12 were compared between IVIG-sensitive group and resistance group

**Figure 3:**
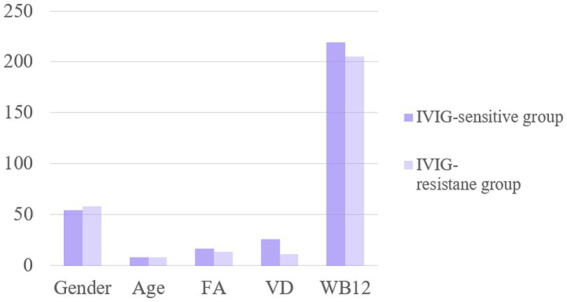
The levels of FA, VD and WB12 indexes were compared between IVIG-sensitive group and resistance group.

There were no significant differences in the sex ratio, serum FA levels and VB12 levels between the two groups (*p* > 0.05). Serum vitamin D3 (VD3) levels were significantly higher in the IVIG-sensitive group compared to the IVIG-resistant group (*p* < 0.05), as shown in Figure 3. Multifactor regression analysis showed that decreased serum VD3 level is an independent risk factor for IVIG resistance (Table 5).

**Table 5:** Multiple regression analysis.

Parameters	β	SE	Wald χ^2^	OR	95%CI	*p*
VB12	1.295	0.335	4.886	2.875	1.202–5.062	0.135
VD3	0.937	0.293	3.133	1.593	0.859–3.686	0.016

VD3, vitamin D3; VB12, vitamin B12.

### 3.5 Comparisons of FA, VD3, and VB12 levels in the CALs group and the non-CALs group

**Figure 4:**
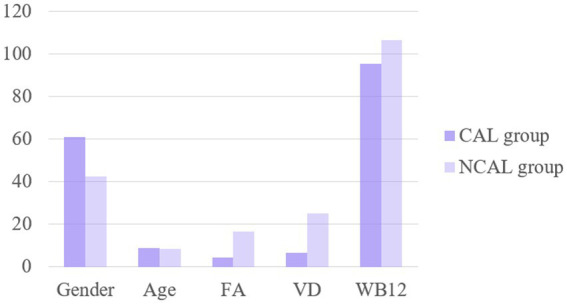
The levels of FA, VD and WB12 in the CAL group and the non-CAL group were compared.

There were no significant differences in sex, age or VB12 levels between the two groups (*p* > 0.05). The serum FA and VD3 levels in the CALs group were significantly lower than those in the non-CALs group (*p* < 0.05), as shown in Figure 4. Multivariate regression analysis showed that FA and VD3 levels are independent risk factors for CALs (Table 6).

**Table 6:** Multiple regression analysis.

Parameters	β	SE	Wald χ^2^	OR	95%CI	*p*
FA	−1.779	0.224	2.117	2.353	0.828–4.025	0.016
VD3	−1.617	0.277	3.736	1.475	0.356–3.053	0.031
VB12	−2.784	0.313	5.883	3.465	1.637–7.371	0.307

FA, folic acid; VD3, vitamin D3; VB12, vitamin B12.

### 3.6 The levels of FA, VD3, and VB12 were compared between small tumor group and medium and large tumor groups

**Figure 5:**
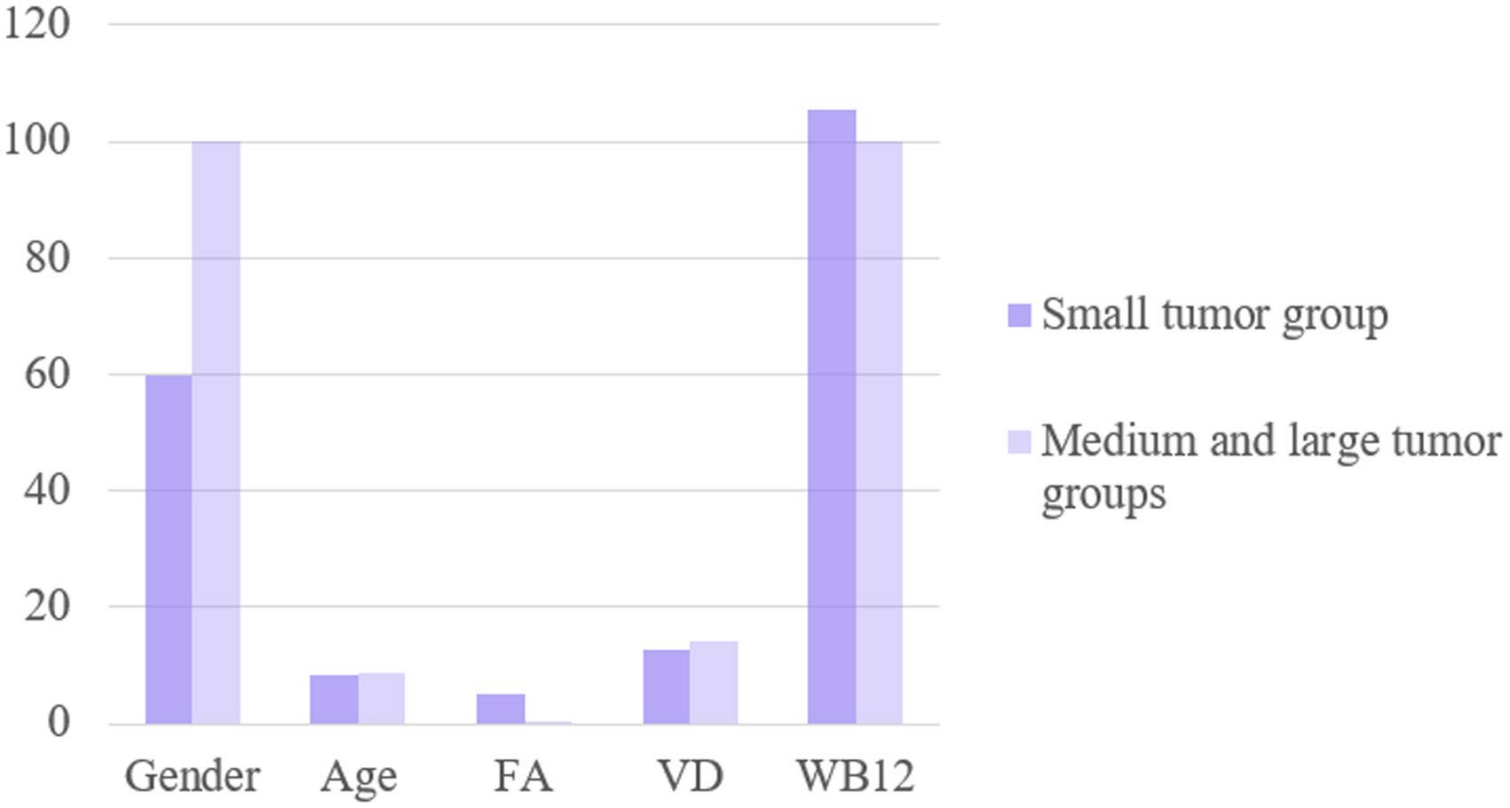
The levels of FA, VD and WB12 were compared between small tumor group and medium and large tumor groups.

There were no significant differences in sex or age between the two groups (*p* > 0.05). The serum FA levels in the small tumor group were significantly higher than those in the medium and large tumor groups (*p* < 0.05), while there was no difference in the VD3 and VB12 levels among these groups (*p* > 0.05), as shown in Figure 5. Multivariate regression analysis showed that the FA level is an independent risk factor for aneurysm size (Table 7).

**Table 7:** Multiple regression analysis.

Parameters	β	SE	Wald χ^2^	OR	95%CI	*p*
FA	−1.002	0.415	3.857	1.742	0.936–3.634	0.009
VB12	−2.373	0.332	5.693	2.216	1.114–5.291	0.552

FA, folic acid; VB12, vitamin B12.

## 4 Discussion

Consistent with previous studies, we observed that serum FA levels in the KD group were significantly lower than those of the healthy group. Specifically, a study conducted by the Japan Environment and Child Research Group (18) showed that insufficient FA intake in pregnant mothers is associated with the occurrence of KD in infants and that FA supplementation in pregnant mothers could reduce the risk of KD. FA serves an essential role as a cofactor in both nucleotide biosynthesis and methylation processes, emphasizing its importance in cellular function and development (19). In addition, Amarasekera et al. (20) reported a positive correlation between FA concentrations in neonatal cord blood and maternal blood. Maternal FA supplementation is associated with upregulation of DNA methylation and genomic imprinting in CD4+ progenitor cells. Furthermore, another study (21) showed that FA inhibited gene expression of inflammatory cytokines such as interleukin-1β, tumor necrosis factor-α, and monocyte chemotactic protein-1 in lipopolysaccharide-activated macrophages. Considering the significant role of these inflammatory cytokines in the pathophysiology of KD, we hypothesize that sufficient FA exposure *in utero* could influence the development of immune functions in offspring, potentially reducing the risk of developing KD. As there was no difference in the estimated daily dietary FA between the KD and healthy groups, further researches are needed to elucidate the mechanism by which FA prevents the onset of KD.

This study additionally revealed that serum FA levels in the IKD group were significantly lower than those in the KD group, and levels in the CALs group were significantly lower than in the non-CALs group. This has not been explicitly mentioned in previous studies, suggesting that FA levels are involved in the occurrence of CALs by influencing Hcy levels. Recent epidemiological studies indicated that Hcy levels are independent risk factors for coronary heart disease. Homocysteine, a sulfur-containing amino acid derived from methionine after demethylation, is synthesized with the assistance of methionine synthase, which requires VB12 as a cofactor (22). This study demonstrated that the serum FA levels in the small tumor group was significantly higher than that in the medium and large tumor groups, while VD3 and VB12 levels did not differ from the other two groups. This deviation from previous studies may be attributed to the limited sample size used in this study. The product of tetrahydrofolate catalyzed by N-5 N-10-methylene tetrahydrofolate reductase (NTHFR) is the most important methyl donor *in vivo*. In addition, *in vivo* Hcy can condense with serine to form cystathione under the catalysis of cystathionine β-synthase (CBS) (23). When FA is deficient, the activities of NTHFR and CBS are significantly reduced, which leads to the obstruction of methionine regeneration and the excessive accumulation of Hcy. In conclusion, FA is an essential component for the conversion of Hcy to methionine and exhibits a significant negative correlation with Hcy levels. A deficiency in FA not only hampers the function of FA reductase but also disrupts the production of methyltetrahydrofolate, contributing to elevated Hcy levels.

The mechanisms by which homocysteine induces coronary artery lesions (CLAs) are complex, involving damage to the vascular endothelium, enhanced platelet activity, increased platelet aggregation, elevated fibrinogen production, and the promotion of smooth muscle cell migration, layering, and other pathophysiological processes (24, 25). These mechanisms bear a resemblance to the pathophysiological processes of CALs in KD; thus, it is hypothesized that homocysteine may also contribute to coronary artery injury in children with KD. Further studies (26) have demonstrated that homocysteine activates protein kinase C, which in turn promotes the expression of c-Fos and c-Myb in vascular endothelial and smooth muscle cells. Fundamental studies (27) have established a strong link between FA, a key factor in Hcy metabolism, and the development of CALs. In the realm of clinical research (28), findings indicated that Hcy levels were significantly higher in CHD patients than in non-CHD patients. Additionally, there is a negative correlation between FA levels and the incidence of CHD, with Hcy levels also showing a negative association with FA levels. However, FA is not an independent risk factor for CHD (29). It has been hypothesized that FA may influence homocysteine (Hcy) metabolism. Furthermore, a deficiency in FA leads to elevated Hcy levels, which, in turn, contributes to the development of CALs and CHD. This study confirmed significant differences in serum VD3 levels across groups: patients in the KD group exhibited lower VD3 levels compared to the healthy control group; within the KD cohort, those with IKD had significantly lower VD3 levels than those with complete KD; and patients with CALs had lower VD3 levels than those without CALs. Additionally, substantial evidence indicated that a deficiency in VD3 elevates the risk of cardiovascular diseases by impairing vascular endothelial function. Recent studies have shown that VD3 levels are related to KD, particularly KD combined with CALs. Research has demonstrated that serum levels of 25-(OH) D3 are significantly lower in children diagnosed with KD and that these levels are positively associated with the incidence of CALs and IKD (30). Furthermore, Stagi et al. (31) also found that a deficiency of serum 25 (OH) D3 in children with KD is closely related to an increased risk of cardiovascular events, and its low level may play an important role in the occurrence of KD combined with coronary complications. In addition, previous studies have found that the serum VD3 level of the population exhibit a seasonal pattern of being lower in winter and higher in summer (32), which is consistent with the seasonal variation trend of KD incidence. Thus, it is hypothesized that the seasonal pattern of KD incidence may be linked to the seasonal fluctuations in serum VD3 levels (33). However, the mechanism underlying the relationship between serum VD3 levels and the pathogenesis of KD remains unclear. Activation of immune factors and the release of inflammatory factors are increasingly recognized as critical elements in KD’s pathogenesis. Experimental studies involving KD mice models have demonstrated overexpression of the transcription factor kappa B (κB) and matrix metalloproteinase-9 (MMP-9) (34). Other literatures also found that interleukin-6 (IL-6), tumor necrosis factor-α (TNF-α), and other inflammatory factors are related to KD. Furthermore, cytokines such as TNF-α, IL-6, and IL-1β have been found to induce endothelial cells to increase the production of matrix metalloproteinases (MMPs) This action accelerates the breakdown of extracellular matrix proteins and basement membrane components, leading to the deterioration of vascular walls and promoting the occurrence of CALs (35). Studies in animal models and cell cultures have revealed that the loss of the VD3R gene can lead to higher levels of pro-inflammatory factors (including TNF-α, IL-6, and IL-β1) produced by bone marine-derived macrophages. Additionally, the signaling pathway generated by the combination of VD3 and VD3R can inhibit the activation of NF-κB, which enhances the negative feedback regulation of the inflammatory response (36). VD3 deficiency leads to an unregulated and persistent inflammatory response, which may also explain the relationship between VD3 deficiency and inflammatory disorders, including KD, to a certain extent (37).

Our study also had some limitations. We measured only plasma folate levels, not its active form, 5-methyltetrahydrofolate (5-MTHF), which we plan to investigate further.

## 5 Conclusion

In summary, serum levels of FA and VD3 were reduced in children with KD. At the same time, it was found that the serum levels of FA and VD3 were particularly decreased in children with CALs, suggesting that the levels of FA and VD3 were involved in the occurrence and development of KD and CALs, providing a new research direction for the pathogenesis of KD and reducing the risk of CALs.

## Data availability statement

The original contributions presented in the study are included in the article/supplementary material, further inquiries can be directed to the corresponding authors.

## Ethics statement

The studies involving humans were approved by Kunming Children’s Hospital, Kunming, Yunnan, China. The studies were conducted in accordance with the local legislation and institutional requirements. Written informed consent for participation in this study was provided by the participants’ legal guardians/next of kin. Written informed consent was obtained from the individual(s), and minor(s)’ legal guardian/next of kin, for the publication of any potentially identifiable images or data included in this article.

## Author contributions

YC: Writing – original draft, Writing – review & editing. XL: Writing – original draft, Writing – review & editing. BL: Formal Analysis, Investigation, Writing – review & editing. JL: Methodology, Writing – review & editing. LM: Conceptualization, Writing – review & editing. CY: Conceptualization, Writing – review & editing. LH: Conceptualization, Writing – review & editing. HL: Project administration, Writing – review & editing. LD: Methodology, Writing – review & editing. ZS: Writing – original draft, Writing – review & editing. XZ: Writing – original draft, Writing – review & editing.
